# Non-European Union doctors in the National Health Service: why, when and how do they come to the United Kingdom of Great Britain and Northern Ireland?

**DOI:** 10.1186/1478-4491-5-6

**Published:** 2007-02-27

**Authors:** Jyothis T George, Kavitha S Rozario, Jeffrin Anthony, Edward B Jude, Gerard A McKay

**Affiliations:** 1 York District Hospital, York, YO31 8HE, United Kingdom of Great Britain and Northern Ireland; 2 Harrogate District Hospital, Harrogate, North Yorkshire, HG2 7SX, United Kingdom of Great Britain and Northern Ireland; 3 Walsgrave Hospital, Coventry, CV2 2DX, United Kingdom of Great Britain and Northern Ireland; 4 Tameside General Hospital, Ashton-under-Lyne, OL6 9RW, United Kingdom of Great Britain and Northern Ireland; 5 Monklands Hospital, Airdrie, ML6 0JS, United Kingdom of Great Britain and Northern Ireland

## Abstract

**Background:**

As many as 30% of doctors working for the National Health System (NHS) of the United Kingdom of Great Britain and Northern Ireland (UK) have obtained their primary qualifications from a country outside the European Union. However, factors driving this migration of doctors to the UK merit continuing exploration. Our objective was to obtain training and employment profile of UK doctors who obtained their primary medical qualification outside the European Union (non-European doctors) and to assess self-reported reasons for their migration.

**Methods:**

We conducted an online survey of non-European doctors using a pre-validated questionnaire.

**Results:**

One thousand six hundred and nineteen doctors of 26 different nationalities completed the survey. Of the respondents, 90.1% were from India and over three-quarters migrated to the UK mainly for 'training'. Other reasons cited were 'better pay' (7.2%), 'better work environment' (7.1%) and 'having family and friends in the UK' (2.8%). Many of the respondents have been in the UK for more than a year (88.8%), with 31.3% having spent more than 3 years gaining experience of working in the NHS. Most respondents believe they will be affected by recent changes to UK immigration policy (86.6%), few report that they would be unaffected (3.7%) and the rest are unsure (9.8%).

**Conclusion:**

The primary reason for many non-European doctors to migrate to the UK is for training within the NHS. Secondary reasons like better pay, better work environment and having friends and family in the UK also play a role in attracting these doctors, predominantly from the Indian subcontinent and other British Commonwealth countries.

## Background

The National Health Service is the public sector organisation providing state-funded healthcare in the United Kingdom and Great Britain. As many as 30% of its doctors have been trained outside the Europe [1]. In some regions, overseas doctors comprise up to 50% of all junior doctors [2]. General Medical Council (GMC), the United Kingdom's regulatory and licensing body for doctors, had a total of 239 661 doctors registered with it on 1 June 2006, 22.8% (n = 54 656) obtained their primary medical qualification outside the European Union.

UK government policy towards non-European Union (non-EU) doctors was changed recently [3]. Non-EU residents normally require a work-permit to take up any employment in the UK. Until recently, doctors in training posts were exempt through a special scheme called permit-free training (PFT). With the new changes to immigration policy, NHS employers wishing to appoint these doctors will have to prove that no suitable European Union applicants are available. This may result non European doctors being unable to compete for NHS jobs. The number of doctors affected by this is estimated to be between ten thousand [4] and sixteen thousand [5]. It is in this context that we undertook the study. Our objective was to provide a self-reported training and employment profile of non-European doctors in the UK and to assess self-reported reasons for their migration.

## Methods

We established an online survey using a pre-validated questionnaire. Validation was carried out with a representative test cohort with feedback from leaders of non-European doctors' organizations. The survey sample was defined as all doctors in the UK who obtained their primary medical qualification outside the EU. Email invitations were sent to various organizations of overseas doctors. Respondents were requested to recommend the survey to other non-EU doctors using a pre-programmed input area on our webpage to ensure maximum reach among our target sample.

Programming tools were used to prevent duplicate submissions and registration numbers with the General Medical Council were used as unique markers.

The survey was open for submission for four consecutive weeks ending 22 April 2006.

## Results

We received one thousand six hundred and nineteen completed responses from doctors of 26 different nationalities. This represents 2.96% of all non-European Union doctors registered with the GMC. Those of Indian nationality represented 90.1%, followed by Pakistan (2.7%), Nigeria (0.9%), Sri Lanka (0.7%), Bangladesh (0.4%) and South Africa (0.4%). All other 21 nationalities added up to a further 4.1%.

The country where respondents obtained their primary qualification mirrors nationality profile. Respondents qualified in India represented 90.1%, 2.8% in Pakistan, 1.1% in Nigeria, 0.5% in South Africa along with 0.4% each in Bangladesh and Sri Lanka. Twelve doctors (less than 0.01%) of non-EU nationality with UK undergraduate medical training also responded to the study. We used these responses in the analysis, but this negligible group of responses does not affect the overall statistics in any meaningful manner.

**Figure 1:**
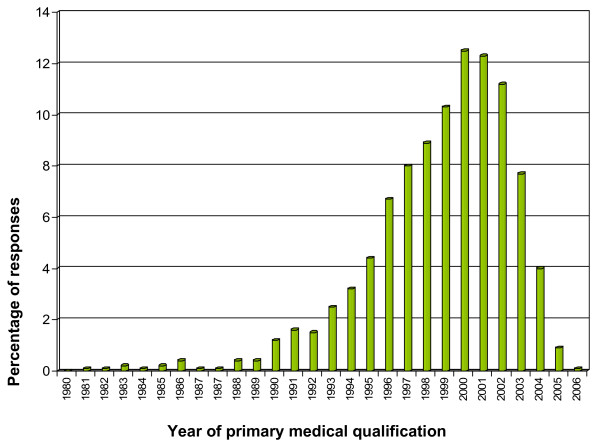
**Diagram showing the year of primary qualification of overseas doctors**. Number of respondents: 1618. Number of respondents qualified prior to 1980: 0.9%.

All respondents were asked to report the year of primary medical qualification. From 1995 onwards, there was a steady rise, with 4.4% qualifying in 1995, 6.7% in 1996, 8% in 1997, 8.9% in 1998, 10.3% in 1999 and 12.5% in 2000. There was a levelling out in 2001 (12.3%) and 2002 (11.2%), but thereafter a drop, with 7.7% qualifying in 2003, 4% in 2004 and 0.9% in 2005 (Figure 1).

**Figure 2:**
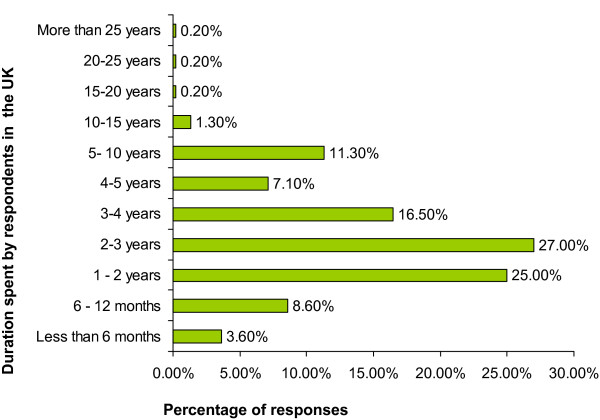
**Diagram showing duration of time spent by overseas doctors in the UK**. Number of respondents: 1617

The respondents were asked to report the duration of time they had spent in the UK. Twenty five percent had been in the UK for more than 1 year but less than 2 years, 27% had spent 2 to 3 years, 16.5% had been in the UK for 3 to 4 years, 7.1% had spent between 4 and 5 years, 11.3% had spent between 5 and 10 years, and 2% had spent more than 10 years. Those who had spent less than one year in the UK amounted to 11.2% (Figure 2).

Of the respondents, 88.9% had held a paid NHS post, while the remaining 11.1% had been unemployed throughout their stay in the UK. Of all the respondents, 12.9% were currently unemployed, suggesting some had failed to secure further employment even after obtaining a paid post in the NHS. At the time of reporting, there were 48% of our respondents employed in junior training posts (foundation trainees, Senior House Officers or House Officers), 17.8% in middle-grade training posts (Specialist Registrar, Locum Appointment for Training) and 11.9% in non-consultant, non-training posts (Staff Grade, Associate Specialists and trust grade doctors). Those employed in research posts totalled 2.5%, while 1.8% were employed as consultants and 1% as GPs. Furthermore, 3.6% were employed in Locum posts and 0.2% have retired from NHS work.

Respondents were asked to report their current immigration status: 39.3% of respondents were on permit-free training; 6.3% were on a visitor's visa; 1.3% of respondents were British Citizens, including 1% who had obtained British Citizenship through naturalisation; 8.5% were working on a work permit; and 37.1% of respondents were on the Highly Skilled Migrant Program or have Permanent Residence (Indefinite Leave to Remain) in the UK.

**Figure 3:**
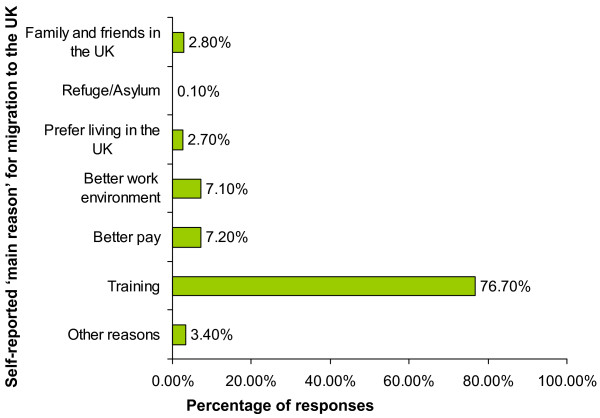
**Main Reason for migration to United Kingdom and Great Britain**. Number of respondents: 1615

All respondents were asked to report their 'main' and 'other' reasons of immigration to the UK (Figure 3). The 'main' reasons for moving to the UK were for training (76.7%), better pay (7.2%), better work environment (7.1%), family and friends in the UK (2.8%) and preference of living in the UK (2.7%). Refuge or asylum seekers amounted to 0.1%, while 3.4% cited 'other reasons'. These include, 'better research opportunities', 'better human rights', 'spouse working in the UK', 'wanted to prove myself amidst the international competition' and 'a step to the USA'.

**Figure 4:**
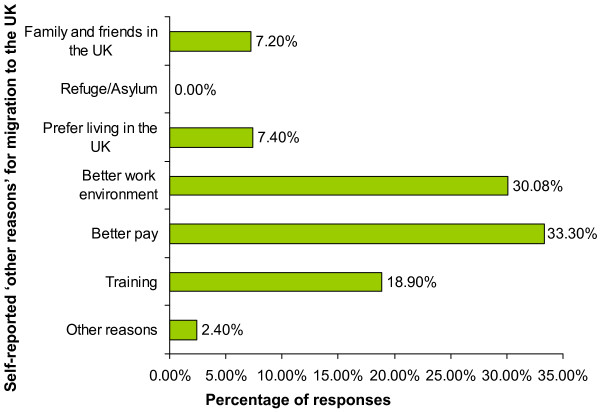
**Other Reason for migration to United Kingdom and Great Britain**. Number of respondents: 1615. Total number of responses: 2150 (multiple responses were allowed and hence the total adds up to >100%)

Among 'other reasons' to move to the UK were better pay (33.3%), better work environment (30.8%), training (18.9%), preference of living in the UK (7.4%) and the presence of family and friends in the UK (7.1%) (Figure 4).

## Discussion

Introduction of the European Working Time Directive, curtailing the working hours of doctors, along with the increased resource investment in the NHS at the end of the last century resulted in an influx of doctors to the UK [6]. Places available for the final part of Professional and Linguistics Board (PLAB test – the General Medical Council's Licensing examination for non-EU Doctors) had to be increased several times to cope with the demand, with the GMC finally opting to set up a custom built examination centre to hold these tests on a daily basis. Also, some NHS trusts had more overseas doctors employed than locally trained graduates [2]. These doctors are younger, more likely to respond to an online survey and are more active in organisations for overseas doctors, especially in the light of changes to UK immigration policy and the Department of Health's employment policy giving preference to EU doctors over their non-European counterparts. These factors, in our view, explain the sample under-representing older Non-European migrant doctors in the UK.

Almost all overseas-trained doctors responding to this survey underwent their training in a commonwealth country or a former British colony. These doctors, took their medical education in English and have successfully demonstrated their English, communication and medical skills by passing the International English Language Testing System (IELTS) and the Professional Linguistic Assessment Board (PLAB) Exam conducted by the General Medical Council.

Our study has two main shortcomings. Firstly, doctors from India are over-represented in our sample cohort. Registration data from the General Medical Council shows a large majority of non-European doctors are from the Indian Subcontinent. As of 1st April 2006, the General Medical Council (GMC) had 22 690 doctors who had qualified in India registered to practice in the UK. Doctors who qualified from all South Asian countries (India, Pakistan, Sri Lanka, Bangladesh and Nepal) add up to 31 302, while all other Non European regions contributed with 21 757 registered doctors. Though every effort was made to reach organisations and forums of doctors of various nationalities, the authors found the response from Indian doctors particularly robust, possibly due to the existence of well-subscribed online groups. It is in this context that one should view the relative over-representation of Indian doctors.

Using an online method of data collection may have limited the reach of this survey. Younger doctors who are more at risk of being affected by changes to immigration policy are more active in organisations representing overseas doctors. More senior doctors as well as others who have spent some time in Britain would therefore be under-represented in our study. Efforts to reach a fully representative sample, though likely to be resource-intensive, would be most welcome.

In an environment of global immigration, doctors have many reasons to migrate and many destinations to migrate to. We believe our data identifies a group of young doctors whose self-reported motivation for migration is assessed here. With evolving immigration policies aiming to manipulate international migration, we believe our data can give valuable insight to workforce planners as well as doctors considering migration.

With the NHS giving preference to EU applicants in employment, it is likely that many of the non-European doctors who are currently in the UK will find it difficult to obtain further training positions to complete their postgraduate training. Resultantly, many may chose to leave the country either to return to their home countries or migrate elsewhere to complete such training.

With increasing competition for training posts and changes to immigration policy [5], many non-European doctors may find it difficult to find employment in the UK and the General Medical Council as well as post-graduate medical education authorities have initiated steps to highlight this fact when in communication with prospective immigrant doctors [6].

## Conclusion

In conclusion, a large majority of non-European doctors in the UK have been attracted by prospects of post-graduate training. Changes to immigration policy that fail to factor in the aspirations and needs of doctors who have already migrated to the UK are likely to disrupt the career paths of many non-European doctors, many of whom have spent a considerable duration of time working and training in the National Health Service of United Kingdom.

## Competing interests

The author(s) declare that they have no competing interests.

## Authors' contributions

JTG conceived the study, analysed results, co-ordinated and prepared the initial manuscript. KSR and JA administered the survey. GAM and EBJ reviewed the literature and edited the manuscript. All authors read and approved the final manuscript.
